# Diabetes Mellitus with Poor Glycemic Control as a Consequence of Inappropriate Injection Technique

**DOI:** 10.1155/2018/7236452

**Published:** 2018-04-01

**Authors:** Ramesh Sharma Poudel, Shakti Shrestha, Sushma Bhandari, Rano Mal Piryani, Shital Adhikari

**Affiliations:** ^1^Hospital Pharmacy, Chitwan Medical College Teaching Hospital, Chitwan, Nepal; ^2^Department of Pharmacy, Shree Medical and Technical College, Chitwan, Nepal; ^3^Department of Internal Medicine, Chitwan Medical College Teaching Hospital, Chitwan, Nepal

## Abstract

Majority of patients with diabetes mellitus (DM), who are on insulin therapy, use insulin pen for convenience, accuracy, and comfort. Some patients may require two different types of insulin preparations for better glycemic control. We have reported a case of poor glycemic control as a consequence of inappropriate insulin injection technique. A 57-year-old man with type 2 DM had been using premix insulin 30 : 70 for his glycemic control for the last 12 years. On follow-up visit, his blood sugar level (BSL) had increased; therefore the treating physician increased the dose of premix insulin and added basal insulin with the aim of controlling his blood sugar level. Despite these changes, his BSL was significantly higher than his previous level. On investigation, the cause of his poor glycemic control was found to be due to inadequate delivery of insulin (primarily premix) as a consequence of lack of priming and incompatibility of single insulin pen for two cartridges. His basal insulin was discontinued and the patient along with his grandson was instructed to administer insulin correctly. After correction of the errors, the patient had a better glycemic control.

## 1. Introduction

Insulin therapy is an effective treatment for controlling blood sugar level (BSL) in type 1 diabetes, gestational diabetes, and certain type 2 diabetes incidences including failure of oral hypoglycemic agents. Sometimes more than one type of insulin is prescribed for better glycemic control. Nowadays insulin pens are preferred for convenience over traditional insulin syringe and vial to inject insulin. Use of pen improves adherence to treatment [1], offers lesser pain during administration [2], and enhances patient confidence in selecting the correct dose of insulin [3, 4]. Correct insulin delivery is critical for better diabetes control [5]. Faulty injection technique not only results in inadequate glycemic control [6] but also results in hypoglycemia [7] and insulin allergy [8, 9]. In this study, we report a case of poor glycemic control as a consequence of inappropriate insulin injection technique.

## 2. Case Report

A 57-year-old man with a 15-year history of type 2 diabetes mellitus visited the outpatient clinic of a hospital for regular check-up. He had been using premix insulin 30 : 70 (Huminsulin 30/70, Lilly Frances S.A.S., 67640 Fegersheim, France) through HumaPen Ergo II (Eli Lilly and Company, Pharmaceutical Delivery System, Lilly Corporate Centre, Indianapolis, IN 46285, USA), 20 units before lunch and 6 units before dinner, to control his BSL for the last 12 years. The patient was also receiving treatment for dilated cardiomyopathy with left ventricle ejection fraction of 20%, hypertension, and chronic kidney disease. On follow-up examination, his fasting (before breakfast) and postprandial (after 2 hours of main meal) BSL were 192 mg/dl and 499 mg/dl, respectively. To control his increased BSL, the treating physician increased the dose of premix insulin 30 : 70 to 28 units in the morning and 14 units in the evening. Additionally, he was advised to use 10 units of basal insulin glargine (Glaritus, Wockhardt Limited, H-14/2 MIDC, Waluj, Aurangabad 431 136), which was from different pharmaceutical company, once daily at 8 pm. Then, physician requested the patient to visit hospital after 2 weeks. On subsequent follow-up visit, BSL of the patient was found to be dramatically high (fasting: 342 mg/dl and postprandial: 554 mg/dl) despite regular use of insulin. The physician requested the patient to get admitted to the hospital in order to control his BSL. However, the patient did not comply with the request, and the physician referred the patient to medication counseling centre of the hospital pharmacy for assessment of insulin injection technique.

**Figure 1:**
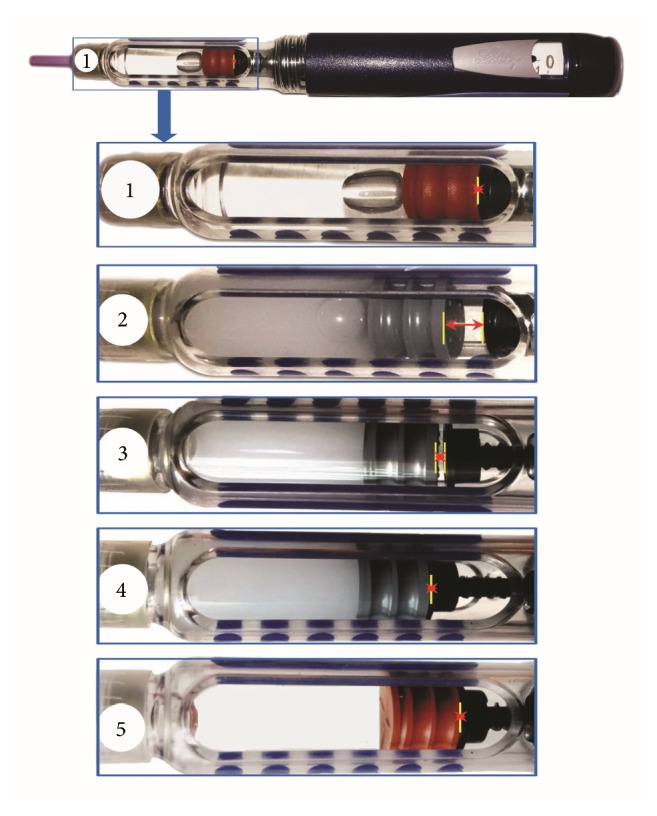
Illustration of insulin delivery during faulty injection technique. Numbers 1–5 demonstrate each of the highlighted steps of the delivery. The cartridge plungers in red and grey are basal and premix (30/70) insulin, respectively. In steps 2 and 3, two yellow lines represent the gap between screw (black) and cartridge plunger (grey) during delivery while a single yellow line represents no gap (steps 1 and 4-5). Details of the figure have been mentioned in the Discussion.

Assessment of his insulin injection technique by a registered pharmacist revealed that the patient was using a single insulin pen (HumaPen Ergo II) for two different insulin preparations (Huminsulin 30/70 and Glaritus) without considering priming and compatibility of the insulin pen for two different cartridges. This resulted in either no release of insulin (steps 2 to 3 in Figure 1) or inadequate delivery of premix insulin 30 : 70 (steps 3 to 4 in Figure 1). Also, the premix insulin was not resuspended by patient or his grandson prior to use and the needle was removed immediately after completely inserting the thumb bottom causing insufficient delivery. There was also lack of knowledge on priming of insulin pen. It was found that the insulin pen was stored in a clay pot. The patient and his grandson were educated about the gap between the screw of insulin pen and cartridge plunger, caused by the use of two different cartridges with different dosages in the same insulin pen without considering priming and compatibility of insulin pen for two cartridges. The counseling pharmacist demonstrated the occasions of no insulin delivery (steps 2 to 3 in Figure 1) and inadequate delivery (steps 3 to 4 in Figure 1) in this case, and his grandson was oriented to appropriate injection technique. Furthermore, the pharmacist also reported the recommending physician about inappropriate insulin injection technique of patient and requested adjusting the dose of insulin. The physician discontinued the basal insulin but advised to continue the premix insulin (28 units in the morning and 14 units in the evening). Four days later, the fasting (before breakfast) and postprandial (after 2 hours of main meal) BSL were found to be 138 mg/dl and 216 mg/dl, respectively. We reinforced the proper injection technique to the patient and his grandson and requested them to visit the hospital after one week for reassessment of the injection technique and determination of fasting and postprandial BSL. Then a plan to adjust the treatment was explained to the patient and his accompanying grandson. Unfortunately, the patient did not visit the hospital for further follow-up. Therefore, we were unable to calculate the mean and standard deviation of fasting and postprandial sugar levels, which require multiple values over a period of time. Hence, the evaluation of the differences over time in terms of statistical significance is not reported. The patient's grandson, on behalf of the patient, has provided written informed consent for the publication of the case report.

## 3. Discussion

Correct insulin injection technique is critical to ensure optimal glycemic control while faulty injection technique can result in inadequate glycemic control, hypoglycemia, and insulin allergy [6–10]. Of all parts an insulin pen consists of a cartridge holder that holds the insulin cartridge, a dose knob to measure the dose, a screw to push the dialed dose of insulin out of the cartridge, a pen needle to inject insulin, and a pen cap to cover the needle and cartrige holder. Some patients may require two different insulin preparations for better glycemic control. Patients who are using two types of insulin preparations need to consider using two insulin pens for injecting the required doses of insulin. While using single pen for two different cartridges, patients need to consider priming before each injection and compatibility of insulin pen for different cartridges. But, in our case, the patient was using a single insulin pen for two different cartridges that were not completely compatible. Moreover, the patient did not consider priming before each injection probably due to lack of proper education on insulin injection technique causing poor glycemic control. The dose knob was initially set at the required units of insulin. On pressing the thumb button the screw inside the insulin pen moved downward to push the cartridge plunger of the cartridge and the dialed dose of insulin was injected through the needle. However, the screw did not retract back to its original position but remained at the position it was initially dialed to. Therefore, subsequent insulin injection from the same cartridge was affected. For this reason, patients need to use two insulin pens for two different cartridges or consider to prime before each injection confirming that single insulin pen is compatible for two different cartridges to inject appropriate dose of insulin. Hence, in our case, due to the variation in the dose of two insulin reparations without priming, the position of the screw was not at the same level corresponding to the level of cartridge plunger in the cartridge (steps 2 and 3 in Figure 1). Subsequently, insulin would not have been injected from the cartridge till the screw would have reached to the level of cartridge plunger in the cartridge. Figure 1 illustrates the insulin delivery through the use of single insulin pen for two different cartridges without considering priming where the patient had used the cartridges of both insulin preparations for 14 days. It can be observed in the figure that the screw was initially tightly attached to cartilage plunger in case of basal insulin with the red cartridge plunger at approximately 140 units (step 1); therefore, the dialed dose of insulin was delivered from the pen. When the cartilage of basal insulin was replaced with premixed insulin in the morning which had cartridge plunger (grey) at approximately 172 units (had used for last 14 days), a gap could be observed between screw and cartridge plunger (approximately 32 units as in step 2). On dialing and pressing 28 units (morning dose) the screw moved 28 units below. However, approximately 4-unit gap still prevailed between the cartridge plunger and screw (step 3), causing no delivery of premix insulin at this time. In the evening 14 units was dialed and pressed. But due to 4-unit gap in step 3, only about 10-unit premix insulin could be delivered (step 4), resulting in insufficient insulin delivery at this time. The cartridge plunger would be moving to approximately 182 units after the evening dose of premix insulin. Similarly, at about 8 pm the premix insulin cartridge was removed and replaced by basal insulin cartridge (plunger with red color). As a result, the screw which had remained at 182 units at the end of evening dose of premix insulin would now move upward and be set at level of approximately 140 units (corresponding to cartilage plunger of basal insulin) and the screw would be tightly attached to cartridge plunger (step 5). On dialing and pressing the 10-unit dose of basal insulin, the screw would move down 10 units to deliver the set dose (insufficient delivery may occur in case of no priming of the pen) and be set at approximately 150 units. Next day premix insulin cartridge (grey) would be replaced by the basal insulin cartridge (red) in the same pen and gaps as shown in step 2 and step 3 would continue to repeat without considering priming.

Although many manufacturers now provide free insulin pens, majority of the patients in our setting need to purchase it due to insufficient distribution and unawareness of patients about the free supply of pens. This encourages patients to use single pen for compatible insulin cartridges to save money. In the present case, the patient was also found to be using the premix insulin without resuspension. A large multicentered study suggests a link between higher consumption of insulin and insufficient mixing of cloudy (premix) insulin prior to use [6]. Patient and his accompanying grandson were trained on the correct insulin technique according to the reference of Forum for Injection Technique and Therapy: Expert Recommendations [11]. Such incidences of errors might be due to lower doctor-to-population ratio in the present healthcare setting of Nepal [12]. Therefore, patient-doctor interaction is restricted due to time limitation [13]. Moreover, the role of pharmacist is usually undermined [14, 15] and majority of community pharmacy professionals have inadequate knowledge and practice on injection technique [16, 17]. Such problem in low-resource setting can be overcome by educating patients through trained comprehensive diabetes educator [18] and subsequent reinforcement and reassessment of insulin injection technique of patients [6].

Our case report highlights the need for continued assessment and education regarding insulin injection technique by trained healthcare professionals, even though the patients are often properly instructed on its correct administration before the initiation of a therapy.

## 4. Conclusion

Use of single insulin pen for two different cartridges without considering priming and their compatibility results in no or inadequate insulin delivery, causing poor glycemic control. Therefore, the healthcare professionals should consider reassessment and reinforcement of insulin injection technique during follow-up visits by patients.

## Data Availability

The data used for the current case report are available from the corresponding author on reasonable request.
